# Staggering the dose of sugammadex lowers risks for severe emergence cough: a randomized control trial

**DOI:** 10.1186/s12871-017-0430-3

**Published:** 2017-10-11

**Authors:** Loh P.S., M.M. Miskan, Chin Y.Z., R.A. Zaki

**Affiliations:** 1 Department of Anesthesiology and Intensive Care, University Malaya, Lembah Pantai, 50603 Kuala Lumpur, Malaysia; 2 Kuching Hospital, Sarawak, Malaysia; 3 Julius Centre University of Malaya, Department of Social & Preventive Medicine, Faculty of Medicine, University Malaya, Kuala Lumpur, Malaysia; 4 Public Health Department, University of Malaya Medical Centre, Kuala Lumpur, Malaysia

**Keywords:** Sugammadex, Reversal, Emergence cough, Emergence agitation, Sore throat

## Abstract

**Background:**

Cough on emergence has been reported as a common adverse reaction with sugammadex reversal. We investigated if staggering the dose of sugammadex will reduce emergence cough in a single-center, randomized, double-blinded study.

**Methods:**

A hundred and twenty ASA 1–3 adults were randomly reversed with 1 mg/kg sugammadex prior to extubation followed by another 1 mg/kg immediately after extubation (staggered group), single dose of 2 mg/kg sugammadex (single bolus group) or neostigmine 0.02 mg/kg with glycopyrrolate (neostigmine group).

**Results:**

We found 70% of patients (*n* = 28) reversed with single boluses of sugammadex had Grade 3 emergence cough compared to 12.5% (*n* = 5) in the staggered sugammadex group and 17.5% (*n* = 7) in the neostigmine group (*p* < 0.001). Besides cough, emergence agitation also appeared highest in the single bolus sugammadex group (*n* = 14, 35%, *p* = 0.005). On the other hand, staggering sugammadex lowered risks of developing severe cough (RR 0.2, p < 0.001) and agitation (RR 0.43, *p* = 0.010) on emergence in addition to cough (RR 0.25, *p* = 0.039) and early sore throat (RR 0.70, *p* = 0.036) in the post-anesthetic care unit. The risks for severe emergence cough (RR 0.86, *p* = 0.762), severe cough in the post-anesthetic care unit (RR 1.0, *p* = 1.000) and sore throat (RR 1.17, *p* = 0.502) were also not different between the staggered sugammadex group and control given neostigmine. In terms of timing, there was no delay in time taken from discontinuing anesthetic agents to reversal and extubation if sugammadex was staggered (emergence time 6.0 ± 3.2 s, *p* = 0.625 and reversal time 6.5 ± 3.5, *p* = 0.809).

**Conclusions:**

Staggering the dose of sugammadex for reversal will effectively decrease common emergence and early postoperative complications.

**Trial registration:**

ANZCTR Number ACTRN12616000116426. Retrospectively registered on 2nd February 2016.

## Background

At the end of surgery, when neuromuscular blockade (NMB) has been used, reversal agents are needed to regain muscle power prior to extubation. With the invention of the first selective relaxant binding agents, sugammadex has completely revolutionized the reversal routine practiced by anesthesiologists all over the world. A general approach is to administer a dose of 2 mg/kg sugammadex to reverse moderate NMB at the end of surgery with higher doses required for deeper depths of NMB or faster reversals [1]. Although it has a fairly safe clinical profile, one of the most commonly reported adverse reactions with sugammadex reversal has been coughing [2]. In a pooled analysis, cough had been reported at least twice as frequently in sugammadex subjects compared to the others treated with placebo [3].

In anesthesia, sudden cough on emergence should be avoided because it can be accompanied by severe laryngospasm or cardiovascular disturbances [4, 5] resulting in post-operative hemorrhage, raised intracranial, intraocular and intra-abdominal pressures [4, 6, 7]. These can lead to detrimental outcomes in a large number of procedures in neurosurgery, thyroidectomy, nasal, eye and spinal surgeries [8, 9]. So far, the postulated mechanism for sugammadex lies in its ability to unmask light anesthesia rapidly resulting in a projected faster recovery of muscle function represented by prevalent cough, movement, grimace or suckling on the tracheal tube upon awakening in the patients [10, 11].

Certainly, many techniques and drugs namely remifentanil and lidocaine have been studied and found to offer protective attributes in avoiding this harmful yet common phenomenon [12–14]. We had not intended to study drugs that suppress emergence cough but wanted to focus on reducing its incidence in patients reversed with sugammadex by comparing a new method of administration to both the conventional way and our old standard, neostigmine. We hypothesized that if sugammadex is given in a staggered dose, the incidence and severity of emergence cough can be reduced compared to the recommended single bolus. Additionally, we also evaluated outcomes in hemodynamic changes during the emergence phase, other recovery profiles and risk factors involved in emergence cough.

## Methods

This was a randomized, double-blinded study conducted in a single tertiary center following approval by the institutional ethics committee (MECID.NO: 20,156–1389) and registration at https://www.anzctr.org.au/Trial/Registration/TrialReview.aspx?id=368609 (ACTRN12616000116426). After obtaining written informed consent, patients aged 18–70 years old with ASA physical status 1 to 3 scheduled to undergo either elective or emergency surgery under general anesthesia requiring rocuronium-induced NMB were included in the study. We excluded candidates who were planned to remain intubated post-operatively for ventilation, tracheostomized, Body Mass Index (BMI) > 40 kg.m^−2^, had evidence of raised intracranial or intraocular pressures, coagulopathy, residual neuromuscular weakness affecting cough and gag, pregnant or had any contraindications to sugammadex (renal failure with creatinine clearance <60 ml/min using the Cockcroft and Gault formula or allergy).

We randomized patients into three groups of 40 patients each using a computerized generated number to receive i) sugammadex in staggered dose of 1 mg/kg prior to extubation and another 1 mg/kg immediately after extubation to give a total of 2 mg/kg ii) sugammadex full dose of 2 mg/kg as a rapid single bolus prior to extubation or iii) neostigmine 0.05 mg/kg in combination with glycopyrrolate 0.01 mg/kg. The assignment was concealed in opaque envelopes to be handed to the attending anesthetist of each case.

In the operating theatre, standard monitoring with oxygen saturation, non-invasive blood pressure, electrocardiogram, end-tidal carbon dioxide partial pressure (ETCO_2_) was applied before induction of anesthesia. An acceleromyography (TOF-Watch**®**; Organon Ltd., Dublin, Ireland) positioned at the adductor pollicis was used to monitor neuromuscular function.

Patients were unpremedicated, received pre-oxygenation in the reverse Trendelenburg position with spontaneous ventilation followed by induction of anesthesia using 1–2 mcg/kg fentanyl, 2–3 mg/kg propofol and rocuronium 0.6–1.0 mg/kg. Intubation was performed using tracheal tubes measuring 7.0 mm internal diameter for women and 8.0 mm for men. Pressures on the high-volume low-pressure cuffs were inflated and kept less than 25 mmHg with a manometer.

For maintenance of anesthesia, either total intravenous anesthesia with propofol and remifentanil or balanced anesthesia techniques with inhalational agents were used according to individual case requirements and management of the attending anesthetist. Mechanical ventilation was performed to achieve a tidal volume of 7 ml/kg aiming for a target of ETCO_2_ 35 - 45 mmHg with air: oxygen mixture at FiO_2_ 0.5. Active measures with forced-air warming blankets were taken to keep patients normothermic and we titrated 0.1–0.2 mg/kg of morphine or target controlled infusion (TCI) remifentanil to effect site concentration of 0.5–3.0 ng/ml as intraoperative analgesics. Boluses of 5 - 10 mg of rocuronium were administered at every 30–40 min as maintenance of moderate NMB in order to achieve train-of-four (TOF) count 1–2.

Thirty minutes prior to the end of surgery, all patients received 40 mg of parecoxib with a dose of anti-emetics, 4 mg ondansetron. Upon completion of surgical skin sutures, careful oral suction and if needed, tracheal tube suction would be performed at this stage and mechanical ventilation switched to pressure support ventilation with a low flow trigger. Care was taken to ensure return of spontaneous breathing efforts that triggered support before the inhalational or intravenous anesthetic agents including remifentanil were discontinued simultaneously. The phase when all anesthetic agents were turned off marked the start of emergence time until reversal was administered. Levels of minimum alveolar concentration (MAC) or propofol effect-site concentration were monitored closely until they dropped to 0.5 and 0.5mcg/ml respectively. The attending anesthetist would begin to intermittently stimulate the patient verbally or with gentle tactile stimulation at intervals of 1–2 min. Should remifentanil be used, its concentration must be below 1.0 ng/ml. Oral or tracheal suction in the emergence phase was avoided and other disturbances such as noise were kept to a minimum.

The attending anesthetist was responsible for the preparation of reversal agents after breaking each concealed assignment. All reversal drugs were delivered discreetly and unseen by a blinded assessor, one of the authors in the group upon completion of surgery, TOF count 1–2, if patients were stable and triggered spontaneous breathing. Similarly, the attending anesthetist and his team managed all tasks related to emergence from anesthesia, emergencies or adverse events arising from the study drug and protocol during this stage. Extubation time defined as time taken from delivery of the reversal drug till tracheal extubation was recorded.

During the emergence phase and reversal period, either authors MM or YZ graded the number and severity of cough: 0 - no cough; 1 - mild, single cough; 2 - moderate, more than 1 lasting for < 5 s; 3 - severe, sustained cough for >5 s or bucking with cough defined as a sudden contraction of the abdomen [8]. Mean arterial blood pressure (MAP) and heart rate (HR) were recorded in the emergence phase until 5 min post-extubation.

When the patients opened their eyes and responded to verbal command, they were encouraged to breathe deeply. The trachea was extubated after confirming spontaneous respiration, adequate tidal volume, respiratory rate and TOF ratio > 0.9 with 6 L/min of oxygen supplemented immediately via a facemask. Sedation levels at 5 min post-extubation were graded with Ramsay Sedation Scale (RSS) [15]. Once stable respiratory and hemodynamic status was ascertained, patients were transported to the post-anesthetic care unit (PACU).

In PACU, other postoperative events at 60 min after extubation were documented. The parameters of interest were defined by the following criteria: severe postoperative cough, grade 3 cough; sore throat, discomfort assessed by visual analogue scale (VAS) more than 5; nausea, and the need for additional anti-emesis; hypertension and tachycardia, an increase of 30% from baseline MAP and HR; agitation, RSS score 1 and desaturation, oxygen saturation less than 95% on facemask. Patients were discharged from PACU once they fulfilled local discharge criteria.

A literature search showed that the only reported incidence of cough with sugammadex in a confluent of signs demonstrating light plane of anesthesia was 20% [10]. Since that study was not designed specifically to identify emergence cough alone nor its grading, we decided to estimate our sample size based on 50% incidence of severe cough as the main outcome in a pilot study with sugammadex 2 mg/kg given conventionally as a single rapid bolus. A sample size calculation using OpenEpi version 3.03 indicated that a sample size of 120 patients with *n* = 40 per arm of the trial was required to detect a difference in cough reduction to 20% with a staggered dose at a power of 80% and α of 0.05.

Data was analyzed using IBM SPSS Statistic 21 with descriptive analysis for baseline data and characteristics of the three groups. Categorical data was analyzed for incidence of outcome in each group by calculating relative risks and then tested using chi-square or Fisher’s exact test where appropriate. All analyses were conducted based on intention-to-treat analysis with *p*-value of 0.05 considered as statistically significant.

## Results

**Fig. 1:**
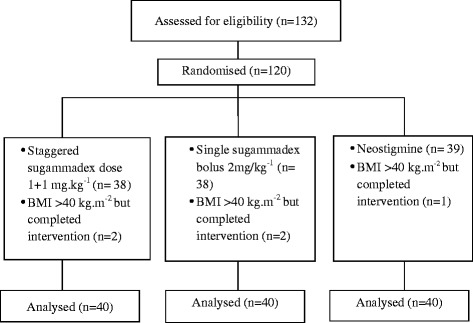
CONSORT diagram demonstrating the attrition numbers in each group

A total of 120 patients were enrolled in this study and randomized into three groups. Five did not fulfill all inclusion criteria with their BMI exceeding 40 kg.m^−2^ but completed the study protocol. With intention-to-treat-analysis, all 120 were studied (Fig. 1).

Patient characteristics and perioperative data were comparable among the groups in Table 1. All patients from the staggered 1 + 1 mg/kg sugammadex and neostigmine groups received balanced anesthesia with inhalational agents, opioids and NMB. Only one patient (2.5%) in the single bolus 2 mg/kg sugammadex group had total intravenous anesthesia using propofol-remifentanil infusion.

**Table 1:** Patient characteristics and perioperative data. Values are mean (SD) or numbers (proportion)

	Sugammadex 1 + 1 mg/kg group *n* = 40	Sugammadex 2 mg/kg group *n* = 40	Neostigmine group *n* = 40
Age; years	46.6 (16.8)	39.5 (14.5)	44.9 (16.5)
Male/Female; n	13/27	24/16	20/20
BMI; kg.m^−2^	25.7 (5.7)	26.1 (6.3)	25.1 (6.1)
ASA I/II/III; n	22/14/4	23/15/2	20/17/3
*Premorbid status*			
Cough; n			
Acute < 2 weeks	1 (2.5)	0	0
Chronic	0	2 (5.0)	1 (2.5)
No cough	39 (97.5)	38 (95.0)	39 (97.5)
Asthma; n			
Controlled	1 (2.5)	4 (10.0)	1 (2.5)
Uncontrolled	0	0	0
Non-asthmatic	39 (97.5)	36 (90.0)	39 (97.5)
Smokers; n			
≥ 10 cigarettes/day	3 (7.5)	9 (22.5)	6 (15.0)
< 10 cigarettes/day	1 (2.5)	1 (2.5)	2 (5.0)
Ex-smoker	3 (7.5)	1 (2.5)	4 (10.0)
Never smoked	33 (82.5)	29 (72.5)	28 (70.0)
*Operative data*			
Types of surgery; n			
General surgery	14 (35.0)	11 (27.5)	11 (27.5)
Neurosurgery	4 (10.0)	3 (7.5)	11 (27.5)
Orthopedic	6 (15.0)	8 (20.0)	5 (12.5)
Gynecology	13 (32.5)	9 (36.0)	3 (7.5)
Ear, nose and throat	0	2 (5.0)	4 (10.0)
Dental	0	2 (5.0)	0
Opthalmology	1 (2.5)	2 (5.0)	0
Urology	2 (5.0)	3 (7.5)	6 (15.0)
Duration of surgery; n			
< 1 h	0	2 (5.0)	3 (7.5)
1-4 h	33 (82.5)	32 (80.0)	30 (75.0)
> 4 h	7 (17.5)	6 (15.0)	7 (17.5)
Anesthesia time; min	165.1 (73.7)	162.2 (81.0)	164.0 (86.7)
Intubation; n			
Normal (CML I-II)	36 (90.0)	34 (85.0)	36 (90.0)
Difficult (CML III-IV)	4 (10.0)	6 (15.0)	4 (10.0)
Rocuronium; mg	54.9 (18.1)	56.9 (19.6)	52.0 (15.3)
Remifentanil; n	4 (10.0)	2 (5.9)	9 (22.5)
Inhalational agent; n			
Sevoflurane	19 (47.5)	18 (45.0)	20 (50.0)
Desflurane	21 (52.5)	21 (51.5)	20 (50.0)
None	0	1 (2.5)	0

*BMI* body mass index, *ASA* American Society Association physical status, *CML* Cormack and Lehane grade

Procedures performed included laparotomy, laparoscopic surgery, mastectomy and thyroidectomy in general surgery; bipolar hemi-arthroplasty, interlocking nail and plating of long bone fractures in orthopedic; open and laparoscopic abdominal hysterectomy in gynecology; craniotomy for excision of brain tumors in neurosurgery; transurethral resection of bladder tumor in urology and direct laryngoscopy and excision under ear, nose and throat surgery. Of these surgeries, 76.7% were performed electively and the rest were emergency cases (23.3%).

During emergence, 28 patients (70%) reversed with single boluses of 2 mg/kg sugammadex coughed severely compared to the other 2 groups with statistical significance of *p* < 0.001 (Table 2). The majority of patients in the staggered 1 + 1 mg/kg sugammadex and neostigmine groups had mild to moderate cough with the total number of 34 (85.0%) and 32 (80.0%) cases respectively. Only 2 (1.67%) cases in all groups combined had no cough during emergence.

**Table 2:** Emergence profiles by group. Values are mean (SD) or numbers (proportion)

	Sugammadex 1 + 1 mg/kg group	Sugammadex 2 mg/kg group	Neostigmine group	*p*-value
	*n* = 40	*n* = 40	*n* = 40	
Emergence time; min	6.0 (3.2)	5.9 (2.5)	6.6 (3.7)	0.625
Reversal time; min	6.5 (3.5)	6.4 (4.5)	6.9 (3.4)	0.809
Emergence cough; n				
No cough	1 (2.5)	0	1 (2.5)	<0.001
Mild (single cough)	17 (42.5)	4 (10.0)	23 (57.5)	
Moderate (lasting < 5 s)	17 (42.5)	8 (20.0)	9 (22.5)	
Severe (sustained > 5 s)	5 (12.5)	28 (70.0)	7 (17.5)	
Sedation level; n				
RSS 1	6 (15.0)	14 (35.0)	3 (7.5)	0.005
RSS 2&3	34 (85.0)	26 (65.0)	37 (92.5)	

Emergence time, time taken from discontinuation of anesthesia till administration of reversal agent; reversal time, time taken from administration of reversal agent until tracheal extubation

*RSS* Ramsay Sedation Scale

**Fig. 2:**
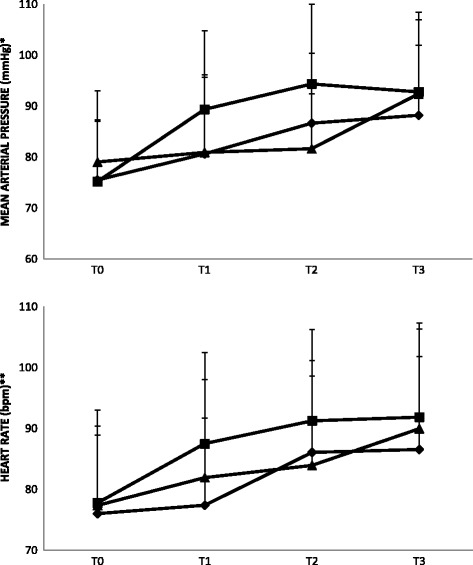
Changes in mean arterial pressure and heart rate during emergence from general anesthesia with sugammadex 1 + 1 mg/kg (♦), sugammadex 2 mg/kg (■) or neostigmine (▲). T0, baseline reading before induction; T1, the end of surgery; T2, immediately before extubation; T3, 5 min after extubation. * *p* = 0.213, ** *p* = 0.123

Time taken for emergence and reversal was not statistically different although both were slightly longer in the neostigmine group. Figure 2 illustrates measurements of mean arterial pressure and heart rate during the emergence phase, which were not statistically different among the groups.

Recovery profiles in PACU are represented in Table 3. Significant difference was found in the proportion of cases among the groups who developed severe postoperative cough (*p* = 0.005) and sore throat (*p* = 0.019) in PACU. The rest of the parameters were not significantly different. There was no reported incidence of re-intubation or unplanned intensive care unit admission.

**Table 3:** Recovery profiles in post-anaesthetic care unit (PACU) by group. Values are mean (SD) or numbers (proportion)

	Sugammadex 1 + 1 mg/kg group	Sugammadex 2 mg/kg group	Neostigmine group	*p*-value
	*n* = 40	*n* = 40	*n* = 40	
Postoperative cough; n	3 (7.5)	12 (30.0)	3 (7.5)	0.005^*^
Sore throat; n	21 (52.5)	30 (75)	18 (45)	0.019
Nausea and vomiting; n	6 (15.0)	6 (15.0)	6 (15.0)	1.000^*^
Hypertension; n	2 (5.0)	3 (7.5)	5 (12.5)	0.601^*^
Tachycardia; n	3 (7.5)	4 (10.0)	3 (7.5)	1.000^*^
Desaturation; n	1 (2.5)	2 (5.0)	0	0.772^*^
Agitation; n	4 (10.0)	3 (7.5)	2 (5.0)	0.773^*^

^*^Fisher exact test

Table 4 represents comparison of relative risks for several main outcomes in this study between groups during emergence and while recovering in PACU. Patients given boluses of 2 mg/kg sugammadex were at least 4 times more likely to develop severe emergence cough, agitation on awakening and post-operative cough in PACU than patients given the standard reversal dose of neostigmine. The group of patients given sugammadex 2 mg/kg in single bolus will also have higher risks of developing early sore throat in PACU. Staggering sugammadex to 1 + 1 mg/kg lowered risks for severe emergence cough, emergence agitation, severe postoperative cough and early sore throat in PACU compared to those given 2 mg/kg sugammadex. No statistical difference was seen in the relative risks for these outcomes when the staggered 1 + 1 mg/kg sugammadex group was compared to the neostigmine group.

**Table 4:** Analyses of main outcomes during emergence and recovery. Values are numbers (proportion)

	Sugammadex 1 mg + 1 mg/kg group *n* = 40	Neostigmine group *n* = 40	RR (95% CI)	*p*-value
Emergence of severe cough				
Yes	5 (12.5)	7 (17.5)	0.71(0.25–2.06)	*p* = 0.531
No	35 (87.5)	33 (82.5)		
Emergence agitation				
Yes	6 (15.0)	3 (7.5)	2.00 (0.54–7.45)	*p* = 0.480
No	34 (85.0)	37 (92.5)		
Cough in PACU				
Yes	3 (7.5)	3 (7.5)	1.00 (0.21–4.66)	*p* = 1.000
No	37 (92.5)	37 (92.5)		
Sore throat				
Yes	21 (52.5)	18 (45.0)	1.17 (0.74–1.83)	*p* = 0.502
No	19 (47.5)	22 (55.0)		
	Sugammadex 2 mg/kg group *n* = 40	Neostigmine group *n* = 40	RR (95% CI)	p-value
Emergence of severe cough				
Yes	28 (70.0)	7 (17.5)	4.00 (1.98–8.08)	p < 0.001
No	12 (30.0)	33 (82.5)		
Emergence agitation				
Yes	14 (35.0)	3 (7.5)	4.67 (1.45–15.00)	*p* = 0.003
No	26 (65.0)	37 (92.5)		
Cough in PACU				
Yes	12 (30.0)	3 (7.5)	4.00 (1.22–13.11)	*p* = 0.010
No	28 (70.0)	37 (92.5)		
Sore throat				
Yes	30 (75.0)	18 (45.0)	1.67 (1.13–2.45)	*p* = 0.006
No	10 (25.9)	22 (55.0)		
	Sugammadex 1 mg + 1 mg/kg group *n* = 40	Sugammadex 2 mg/kg group *n* = 40	RR (95% CI)	*p*-value
Emergence of severe cough				
Yes	5 (12.5)	28 (70.0)	0.21 (0.10–0.45)	*p* < 0.001
No	35 (87.5)	12 (30.0)		
Emergence agitation				
Yes	6 (15.0)	14 (35.0)	0.43 (0.18–1.00)	*p* = 0.039
No	34 (85.0)	26 (65.0)		
Cough in PACU				
Yes	3 (7.5)	12 (30.0)	0.25 (0.08–0.82)	*p* = 0.010
No	37 (92.5)	28 (70.0)		
Sore throat				
Yes	21 (52.5)	30 (75.0)	0.70 (0.50–0.99)	*p* = 0.036
No	19 (47.5)	10 (25.0)		

*PACU* post-anaesthetic care unit, *RR* relative risk, *CI* confidence interval

When analyzed, three risks factors for severe cough on emergence were identified using multinomial regression analysis. Active, passive or even ex-smokers were at higher risk of developing severe emergence cough (OR: 8.1, 95% CI: 3.2–20.0, *p* < 0.001). Similarly, a strong association was found between male gender and severe emergence cough (OR: 8, 95% CI: 3.3–19, p < 0.001). Given as a single bolus, Sugammadex 2 mg/kg group was the third risk factor associated with severe cough on emergence (OR: 3.5, 95% CI: 1.4–8.8, *p* = 0.006). Higher risks were seen in those who were less than 65 years old although statistically not significant (OR: 3.8, 95% CI: 0.8–17.9, *p* value = 0.06) while other predicted risk factors like bronchial asthma, pre-morbid cough and difficult intubation were not significant risk factors according to our findings.

## Discussion

Our study demonstrated that staggering the dose of sugammadex by administering 1 mg/kg at reversal and another 1 mg/kg immediately upon tracheal extubation had significantly less severe emergence cough than administering sugammadex in a single bolus of 2 mg/kg. Significant reductions were also demonstrated in other outcomes during emergence and recovery in PACU in the group given a staggered dose of sugammadex.

Despite having used sugammadex for years and being aware that emergence cough is described as a common adverse effect in its drug information, evidence that looks at its incidence, mechanism and prevention is still lacking [1]. The reported incidence of cough in patients reversed with sugammadex varies from 2.8 to 20.4% in studies done nearly a decade ago [3, 10]. 70% of our patients reversed with a single bolus of 2 mg/kg sugammadex developed severe emergence cough. This was unexpectedly high considering only Grade 3 cough, following the definition published in a previous study done by Lee, was selected and not mild to moderate cough [8]. This finding may be the true reflection of the actual incidence compared to the estimate quoted by Sparr et al. who had previously included all symptoms of light anesthesia and not cough by itself [10]. However, the most likely explanation for such a phenomenon remains the same as the result of its indirect effect attributed to light anesthesia after the fast removal of one limb, NMB in an otherwise balanced technique [2].

Knowledge that the incidence may not be negligible after all, places greater importance for research work to explore safer methods that can avoid such an untoward incidence when using sugammadex as the reversal agent especially when it is strongly indicated to be a better option [16]. Staggering the dose to 1 mg/kg at reversal followed by another immediate dose of 1 mg/kg sugammadex upon extubation reduces the risk of developing severe emergence cough by a fifth compared to the conventional method of a single bolus of 2 mg/kg in our study. In fact, the proportion will then be comparable to the control group given neostigmine for reversal, although not nil but nevertheless lower at 15–17.5%. This finding has not been previously represented in any other studies.

Besides emergence cough, reduction in risks for agitation during extubation was also significantly decreased in the group given sugammadex 1 + 1 mg/kg. Patients given sugammadex 2 mg/kg in single boluses were 4.67 times more likely to develop agitation on emergence than those reversed with neostigmine and 2.33 times more likely than sugammadex 1 + 1 mg/kg. Agitation together with coughing form the signs of light anesthesia that were first described as the main side effects for sugammadex in clinical practice since 2009 [2]. The parallel drop in both clinical effects further confirms that the return of muscle power will be a gradual event if sugammadex is staggered.

More than 98% of our cases developed cough on emergence in varying degrees of severity. As the depth of anesthesia decreases after discontinuing anesthetic agents, the probability of coughing increases due to constant larnygotracheal stimulus from the tracheal tube [17]. In current literature, the overall incidence in the population undergoing general anesthesia varies greatly from study to study. For example, a study by Kim et al. in 1998 quoted 52 patients out of 68 (76%) coughed before responding to command. A similar study by Aouad et al. using the same anesthetic agents as ours for induction, maintenance and reversed with neostigmine in the control arm had 24 out of 30 (80%) patients coughing in the presence of a tracheal tube [13]. The present result for overall emergence cough closely resembles a third report with estimates of 96% coughing as an end-point of tracheal-tube-induced emergence phenomenon [14]. The difficulty in estimating the risk of an anesthetized population and its large variability lie in the inability to create a completely standardized condition at different time points during the wake-up period when the frequency and severity of cough is being determined [17]. These were taken into consideration when we attempted to standardize the emergence routine of all cases but put in clinical perspectives, emergence conditions cannot be exactly the same for each and every one of the patients.

Several anesthetic agents and techniques such as opioids like remifentanil continued at effect site concentration of 2 ng/ml until extubation have antitussive effects through their modulation within the central nervous system [12, 18]. Hence, at reversal the concentration below 1.0 ng/ml at effect site was required. Similarly, a high dose of lidocaine or intracuff lidocaine will suppress the cough reflex by inhibiting the formation of action potentials in tracheal cough receptors [18–20]. Total intravenous anesthesia (TIVA) has also demonstrated significantly less coughing compared to balanced anesthesia technique [17] but out of 120 cases recruited in our study, only one (0.8%) had TIVA, 15 (12.5%) had remifentanil which was discontinued at the end and none had lidocaine in the tracheal cuff or lidocaine sprayed at the vocal cords. Therefore, coughing was not unexpectedly high in our study at 98% compared to all the other published reports. In clinical practice, TIVA and TCI remifentanil maybe feasible in surgical procedures where any slight bucking or movement can be detrimental in addition to staggering the dose of sugammadex as reversal.

Our results also identified two other significant risk factors for emergence coughing namely gender, males had a higher preponderance to coughing and secondly, smoking as well regardless of whether they were ex-smokers or currently still smoking. This is similar to the finding by Hans et al. on smokers versus non-smokers awakening from general anesthesia at the end of cervical spine surgery [9]. Gender differences in requirements for preventing cough during anesthetic emergence had been investigated and may point to a higher cough sensitivity in males than females [21]. In addition, we found that age of less than 65 years old may also be a risk factor for cough on emergence.

In terms of timing, both emergence and reversal times were slightly longer in the neostigmine group compared to the other two as expected, although not statistically significant. But more importantly, we could deduce that a mean duration of 6.5 min (±3.5) between the first dose of 1 mg/kg sugammadex and the second dose did not cause any delay to extubation when compared to sugammadex 2 mg/kg with a mean reversal time of 6.4 (±4.5), *p* = 0.809.

In PACU, coughing and sore throat were significantly higher with sugammadex 2 mg/kg bolus compared to the staggered sugammadex 1 + 1 mg/kg and control neostigmine. Our results for sore throat, cited as one of the most undesirable postoperative morbidities, were 45% in neostigmine and 52.5% in sugammadex 1 + 1 mg/kg. Both results mirror the overall approximate of 50% or more cases with sore throat in the surgical population [22]. This symptom may result from mucosal injury causing inflammation during the process of airway instrumentation [23] and when it occurs immediately, it is primarily due to actions undertaken in the process of extubation [24]. Correlation is shown here where the rate of postoperative sore throat (75%) and severe cough (30%) in the recovery rises with severe emergence cough on extubation seen in sugammadex 2 mg/kg group. Therefore, it seems most plausible that severe coughing with a tracheal tube in-situ just before removal results in a considerable amount of mucosal injury leading to sequelae that last longer than the sudden surge of hemodynamics experienced on table.

Although the results from this study has shown a few unfavorable outcomes when sugammadex was given as a single bolus compared to the traditionally administered neostigmine, we would emphasize that these emergence and early postoperative risks should not downplay the indications for using sugammadex in the first place. As patients’ safety comes first, if clinically indicated, sugammadex at the appropriate dose for the corresponding depth of NMB will reduce incidence of postoperative residual NMB and avoid associated issues with the neostigmine and anti-cholinergic pairing [25]. And again, even though newer studies are beginning to look at cost saving strategies for sugammadex, our study is not about determining its cost reduction, which ultimately should not outweigh good clinical practice in our work [26].

A major limitation in this study was the lack of examination in the exact depth of block when the first staggered dose of sugammadex was given or the depth at the second dose. Therefore, the objective measurement of gradual recovery in neuromuscular function could not be proven other than clinically observed findings, which could possibly influence the ability to cough. Secondly, although studies have shown that reversal with sugammadex eliminated residual NMB and associated clinical symptoms of partial paralysis [27], we could not safely conclude that the duration added between the two doses had differing effects on neuromuscular function after the emergence period. Lastly, a single observer graded the severity of cough in each case without further cross-examination. Perhaps with both present at reversal and by comparing their scores, observer bias could be minimized.

## Conclusion

We found that staggering the dose of sugammadex by giving half of it at reversal and the second half immediately upon extubation, allows a gradual recovery of NMB that most importantly, reduces risks for severe cough and agitation on emergence besides lowering the incidence for early post-operative morbidities such as cough and sore throat in PACU.

## Abbreviations

BMI: Body mass index
CI: Confidence interval
ETCO_2_: End-tidal carbon dioxide partial pressure
HR: Heart rate
MAC: Minimum alveolar concentration
MAP: Mean arterial blood pressure
NMB: Neuromuscular blockade
OR: Odds ratio
PACU: Post-anesthetic care unit
RSS: Ramsay sedation scale
TCI: Target controlled infusion
TIVA: Total intravenous anesthesia
TOF: Train-of-four
VAS: Visual analogue scale
